# *Giardia duodenalis* in Rodents: A Global Systematic Review and Meta‐Analysis

**DOI:** 10.1002/vms3.70546

**Published:** 2025-08-12

**Authors:** Tahereh Davoodi, Jalil Feizi, Kambiz Karimi, Mohammad Reza Mohammadi, Ali Pouryousef, Esfandiar Azizi, Ali Asghari, Fariba Shadfar

**Affiliations:** ^1^ Department of Medical Science Kaz.C., Islamic Azad University Kazerun Iran; ^2^ Zoonotic Diseases Research Center Ilam University of Medical Sciences Ilam Iran; ^3^ Department of Internal Medicine, School of Medicine Ilam University of Medical Sciences Ilam Iran; ^4^ Department of Medical Parasitology and Mycology, School of Medicine Shiraz University of Medical Sciences Shiraz Iran; ^5^ Department of Bacteriology, Faculty of Medical Sciences Tarbiat Modares University Tehran Iran; ^6^ Leishmaniasis Research Center Sabzevar University of Medical Sciences Sabzevar Iran; ^7^ Department of Immunology, School of Medicine Ilam University of Medical Sciences Ilam Iran; ^8^ Medical Microbiology Research Center Qazvin University of Medical Sciences Qazvin Iran; ^9^ Department of Dermatology, School of Medicine Ilam University of Medical Sciences Ilam Iran

**Keywords:** assemblage, Giardia duodenalis, meta‐analysis, molecular prevalence, rodents, systematic review

## Abstract

**Background:**

*Giardia duodenalis* (also known as *G. lamblia* or *G. intestinalis)* is a globally distributed protozoan with zoonotic potential. This systematic review and meta‐analysis aimed to determine the global molecular prevalence and genotypic distribution of *G. duodenalis* in rodents, based exclusively on studies using molecular diagnostic techniques.

**Methods:**

A comprehensive literature search up to 15 October 2024, identified 23 eligible studies encompassing 54 datasets and 5971 rodent samples from 10 countries across three continents. Prevalence estimates were pooled using a random‐effects model, and heterogeneity was assessed via the I^2^ statistic. Assemblage and sub‐assemblage distributions were analysed across rodent species and geographic regions.

**Results:**

The pooled molecular prevalence of *G. duodenalis* in rodents was 7.4% (95% CI: 4.8–11.4%), with chinchillas (36.9%) and porcupines (23.1%) showing the highest infection rates. Rodents were found to harbour six assemblages (A–E, G) and four sub‐assemblages (AI, AII, BIII, BIV) of *G. duodenalis*, with marked geographic variation. The highest pooled prevalence was observed in Europe (17.9%; 95% CI: 9.8–30.5), where assemblages C, D, G, and most occurrences of E, B, and A were reported. Assemblages C and D were entirely absent in Asia. In contrast, most reports of the rodent‐specific assemblage G originated from Asia. South America (represented solely by Brazil) reported only assemblage A. China contributed the largest dataset (*n* = 25) and sample size (*n* = 4009), exhibiting high genetic diversity (A, B, E, G). Belgium also showed notable diversity (A, B, C, E), with assemblage B being the most prevalent in both countries. Assemblage D was found exclusively in Romania, while assemblage C was reported only in Belgium and Italy. Notably, the highest assemblage diversity was observed in chinchillas (five: A–E), squirrels (four: A, B, E, G), and rats (three: A, B, G).

**Conclusions:**

Although various rodent species, especially chinchillas, mice, porcupines, rats, squirrels, and voles, carry *G. duodenalis* zoonotic assemblages (A and B), the overall molecular prevalence in rodents remains relatively low. Due to significant limitations in sampling design, methodological heterogeneity, limited ecological data, and unknown host health status, current evidence is insufficient to confirm rodents as major zoonotic reservoirs. Standardised, large‐scale molecular studies are needed to clarify the epidemiological role of rodents in *G. duodenalis* transmission.

## Graphical abstract

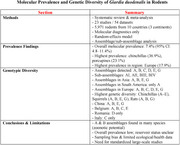

## 1 Introduction

*Giardia duodenalis* (also known as *G. lamblia* or *G. intestinalis*) is a flagellated protozoan parasite that colonises the gastrointestinal system of various mammalian species, including humans (Adam 2001). It is considered one of the primary protozoan agents responsible for parasitic diarrhoea globally and has been categorised as a neglected disease by the World Health Organization due to its substantial impact on public health, especially in low‐resource regions (Savioli et al. 2006; Hatam‐Nahavandi et al. 2025).

This parasite is widely recognised for causing numerous foodborne and waterborne disease outbreaks across the globe, with the extent of infection varying significantly among different regions. Its transmission mainly occurs through the faecal‐oral route, typically via ingestion of food or water contaminated with cysts, or through direct contact with infected individuals or animals (Mohammed Mahdy et al. 2008; Ayed et al. 2024).

Sequence investigations of a number of genetic markers, such as small‐subunit rRNA (*SSU rDNA*), beta‐giardin (*bg*), triosephosphate isomerase (*tpi*), and glutamate dehydrogenase (*gdh*), revealed that *G. duodenalis* is classified into at least eight genetic subgroups (assemblages A‐H), each of which has distinctive host preferences (Huey et al. 2013; Wang et al. 2014; Yu et al. 2019; Heng et al. 2022; Tijani et al. 2023). Humans and other mammals are home to zoonotic assemblages A and B; canids, hoofed animals, cats, rodents, and pinnipeds are home to assemblages C/D, E, F, G, and H, respectively (Ryan et al. 2021). Moreover, rodents can harbour distinct species such as *G. muris*, *G. microti*, and *G. cricetidarum*, which can be differentiated from *G. duodenalis* (Ryan and Zahedi 2019; Argüello‐García and Ortega‐Pierres 2021; Lalle and Caccio 2023).

Among the diverse range of animal hosts, rodents pose a notable public health concern due to their widespread presence, frequent proximity to human dwellings, and remarkable ability to thrive in both urban and agricultural settings. Rodent species such as *Rattus* spp., *Mus musculus*, porcupines, squirrels, voles, and chinchillas have been repeatedly linked to environmental contamination and the zoonotic spread of various pathogens, including *G. duodenalis*. These animals can serve either as silent reservoirs or mechanical carriers, excreting cysts into the environment and promoting transmission via direct interaction or through the contamination of water supplies (Gherman et al. 2018; Helmy et al. 2018; Coppola et al. 2020; Asghari et al. 2022; Rezaie et al. 2025).

Managing diseases transmitted by rodents remains a significant challenge, largely due to their rapid reproduction, behavioural adaptability, and the difficulty of accessing many of their habitats, especially in peri‐urban slums, agricultural settings, and areas where human environments intersect with natural ecosystems (Parsons et al. 2020; Dalecky et al. 2024; Shehata et al. 2025). Therefore, understanding the diversity of *G. duodenalis* genotypes in rodents is critical for evaluating their role in zoonotic transmission and informing effective public health interventions. Hence, the primary objective of this study was to systematically review and quantitatively synthesise the global prevalence and genotypic distribution of *G. duodenalis* in rodent populations using molecular diagnostic methods. By focusing exclusively on molecular studies that specifically identified *G. duodenalis*, this study aimed to provide a comprehensive understanding of the host‐parasite relationship, zoonotic potential, and geographical patterns of assemblages/sub‐assemblages in rodents.

## 2 Methods

### 2.1 Ethics Approval

This study was approved by the Ethics Committee of Qazvin University of Medical Sciences, Qazvin, Iran (Approval No. IR.QUMS.REC.1403.329).

### 2.2 Study Design

A global systematic review and meta‐analysis were conducted to determine the molecular prevalence and distribution of *G. duodenalis* assemblages and sub‐assemblages in rodent populations. The study adhered to the Preferred Reporting Items for Systematic Reviews and Meta‐Analyses (PRISMA) guidelines (Moher et al. 2015).

### 2.3 Search Procedure

An extensive literature search was performed in four international databases: Medline/PubMed, ProQuest, Scopus, and Web of Science, up to 15 October 2024. Google Scholar was utilised to identify relevant grey literature. The search strategy employed the following keywords: (ℌIntestinal Parasitesℍ OR ℌParasitic Infectionsℍ OR ℌ*G. duodenalis*ℍ OR ℌ*G. lamblia*ℍ OR ℌ*G. intestinalis*ℍ OR ℌGiardiasisℍ) AND (ℌPrevalenceℍ OR ℌEpidemiologyℍ OR ℌFrequencyℍ OR ℌOccurrenceℍ) AND (ℌGenotypeℍ OR ℌGenotypingℍ OR ℌAssemblageℍ OR ℌSub‐assemblageℍ) AND (ℌAnimalsℍ OR ℌSmall Mammalsℍ OR ℌRodentsℍ). Additional keywords were applied where necessary, and reference lists of relevant studies were manually screened for further eligible articles. Duplicate records were automatically removed using EndNote X7 software. Two independent reviewers assessed each article for eligibility.

### 2.4 Inclusion/Exclusion Criteria

Only original research articles that used molecular methods (such as PCR‐based techniques) for the detection and genotyping of *G. duodenalis* in rodents were included in this study. To be eligible, studies had to report the prevalence and/or genotypic characterisation (assemblages/sub‐assemblages) of *G. duodenalis* and provide sufficient data for inclusion in the meta‐analysis. Studies based solely on microscopic, immunological, or serological techniques without molecular confirmation were excluded. Additionally, studies that focused on *Giardia* species other than *G. duodenalis* were excluded, even if they used molecular techniques. In molecular studies/datasets involving multiple *Giardia* species, only data related specifically to *G. duodenalis* were considered. Studies that examined non‐rodent hosts or presented data on mixed host species without separate analysis for rodents were also excluded. Review articles, case reports, conference abstracts, and articles lacking sufficient data for molecular prevalence estimation were not included in this study.

### 2.5 Quality Assessment and Data Extraction

The quality of the included studies was evaluated using the Joanna Briggs Institute (JBI) Critical Appraisal Checklist for prevalence studies (Munn et al. 2014). Articles with scores of 4–6 and ≥ 7 were categorised as medium‐ and high‐quality studies, respectively. Key information was extracted independently by two researchers and cross‐validated by other team members. Extracted data included the first author's surname, rodent species, assemblage/sub‐assemblage types, quality assessment scores, year of publication, continent, country, World Health Organization (WHO) region, total sample size, and the number of positive samples.

### 2.6 Meta‐Analysis

Statistical analyses were conducted using Comprehensive Meta‐Analysis (CMA) software, version 3. A random‐effects model was applied to calculate the pooled molecular prevalence and corresponding 95% confidence intervals (CIs) for *G. duodenalis* in rodents. Subgroup analyses were performed based on sample size, continent, country, WHO region, and rodent species. Forest plots were used to present the pooled molecular prevalence along with 95% CIs. Heterogeneity across studies was quantified using the I^2^ statistic, with values interpreted as low (< 25%), moderate (25–50%), or high (> 50%) heterogeneity. Sensitivity analyses were carried out to assess the robustness of the findings by sequentially excluding individual studies. The genetic diversity of *G. duodenalis* and the distribution of its assemblages and sub‐assemblages were reported descriptively.

## 3 Results

### 3.1 Included Articles

**FIGURE 1:**
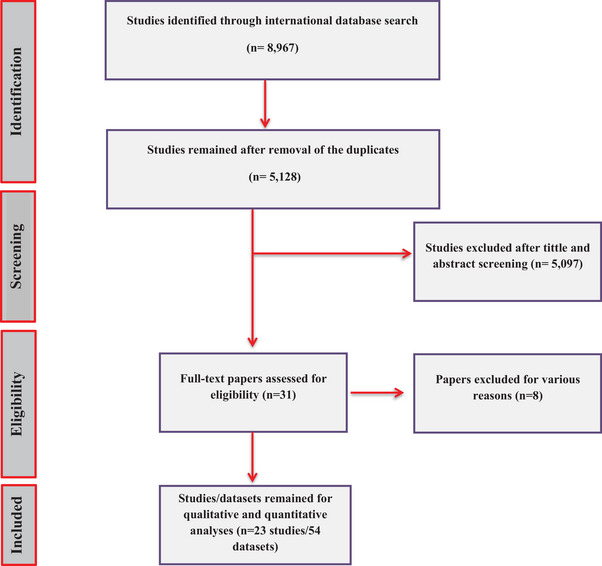
Flowchart depicting the process of included studies in the present systematic review.

Expert researchers conducted a thorough search across four international databases, yielding 8967 initial records. After removing duplicates and reviewing the 5128 remaining papers, 31 articles were selected. A more detailed and meticulous review resulted in the exclusion of eight additional studies, leaving 23 relevant papers (54 datasets) that met the inclusion criteria for this study (Levecke et al. 2011; Veronesi et al. 2012; Fernandez‐Alvarez et al. 2014; Zhao et al. 2015; Gherman et al. 2018; Helmy et al. 2018; Ma et al. 2018; Ma et al. 2024; Deng et al. 2018; Tan et al. 2019; Li et al. 2020; Coppola et al. 2020; Cervero‐Aragó et al. 2021; Cui et al. 2021; Fehlberg et al. 2021; Galán‐Puchades et al. 2021; Galán‐Puchades et al. 2023; Asghari et al. 2022; Wang et al. 2022; Wu et al. 2022; Xu et al. 2022; Zou et al. 2022; Feng et al. 2024) (Figure 1). Studies were excluded due to incomplete or ambiguous results, focus on *Giardia* species other than *G. duodenalis*, or exclusive focus on *G. duodenalis*‐positive cases.

### 3.2 Qualitative and Quantitative Features of the Selected Articles

This systematic review encompassed 23 studies featuring 54 datasets covering the years 2011–2024. The datasets included 26 from rats, 11 from mice, six from squirrels, four from voles, three from chinchillas, two from unidentified rodent species, and one dataset each from guinea pigs and porcupines. The datasets included 25 related to China, nine to Brazil, six to Germany, five to Spain, three to Iran, 2 to Italy, and one each to Austria, Belgium, Malaysia, and Romania. Sample sizes ranged from 1 to 1027 rodent samples, but studies with a sample size of one were excluded from the statistical analysis. Quality assessment with the JBI checklist indicated that nine papers were of high quality (> 6 points) and 14 had moderate quality (4–6 points) (Supplementary Table 1).

### 3.3 Pooled Molecular Prevalence of *G. duodenalis* in Rodents

**FIGURE 2:**
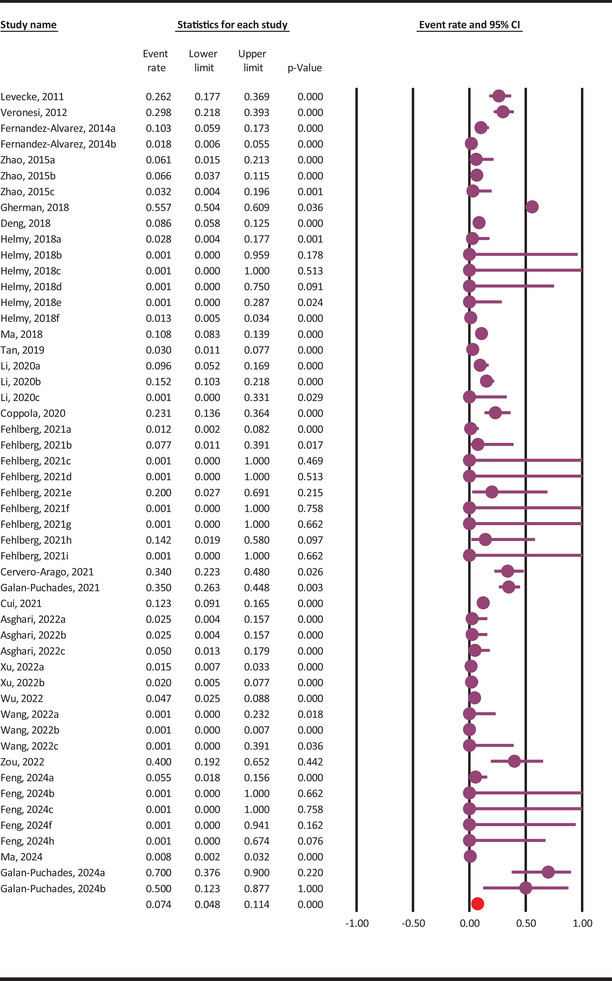
Pooled molecular prevalence of *G. duodenalis* in rodents, with 95% confidence intervals, estimated using a random‐effects model based on included molecular studies.

The pooled molecular prevalence of *G. duodenalis* in rodents was 7.4% (95% CI: 4.8–11.4%), showing substantial heterogeneity among included studies (Q = 618.2, *I^2^* = 91.9%, *p* ≤ 0.001) (Figure 2).

### 3.4 Sensitivity Analysis

**FIGURE 3:**
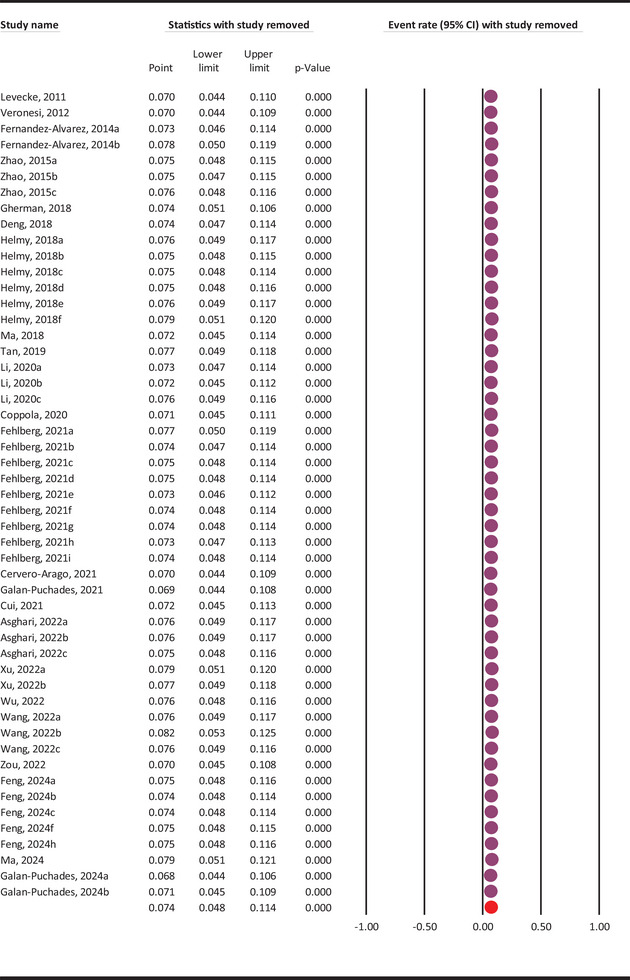
Sensitivity analysis of *G. duodenalis* molecular prevalence in rodents.

The sensitivity analysis revealed that removing individual papers/datasets on *G. duodenalis* infection rates in rodents did not significantly alter the final molecular prevalence (Figure 3).

### 3.5 Pooled Molecular Prevalence of *G. duodenalis* in Rodents Based on Evaluated Subgroups

**FIGURE 4:**
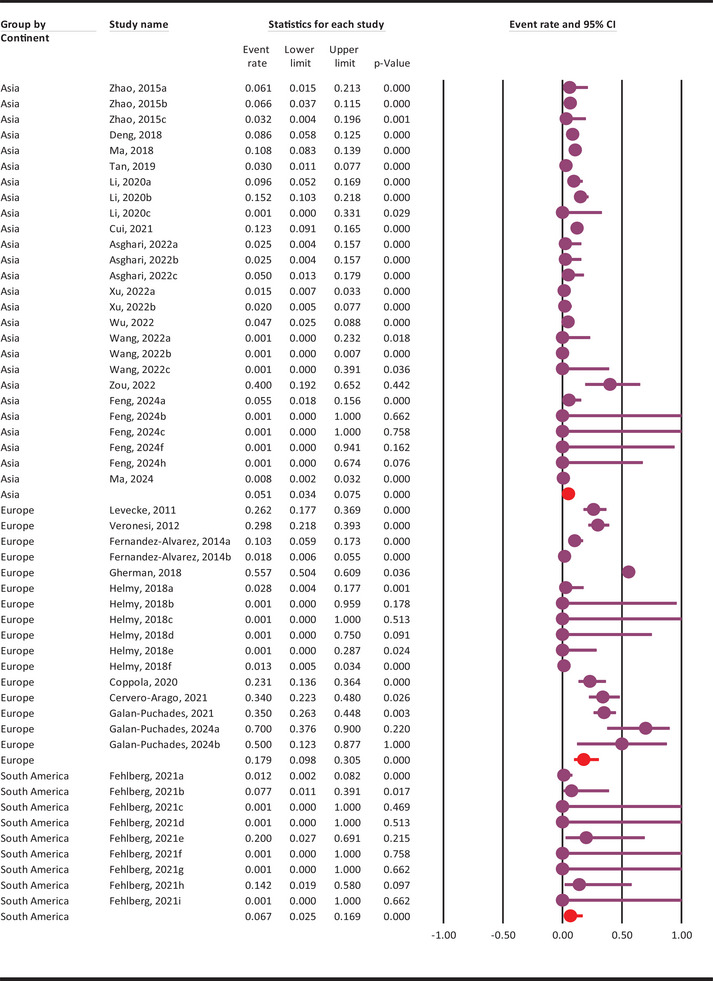
Pooled molecular prevalence of *G. duodenalis* in rodents across continents, with 95% confidence intervals, estimated using a random‐effects model.

**FIGURE 5:**
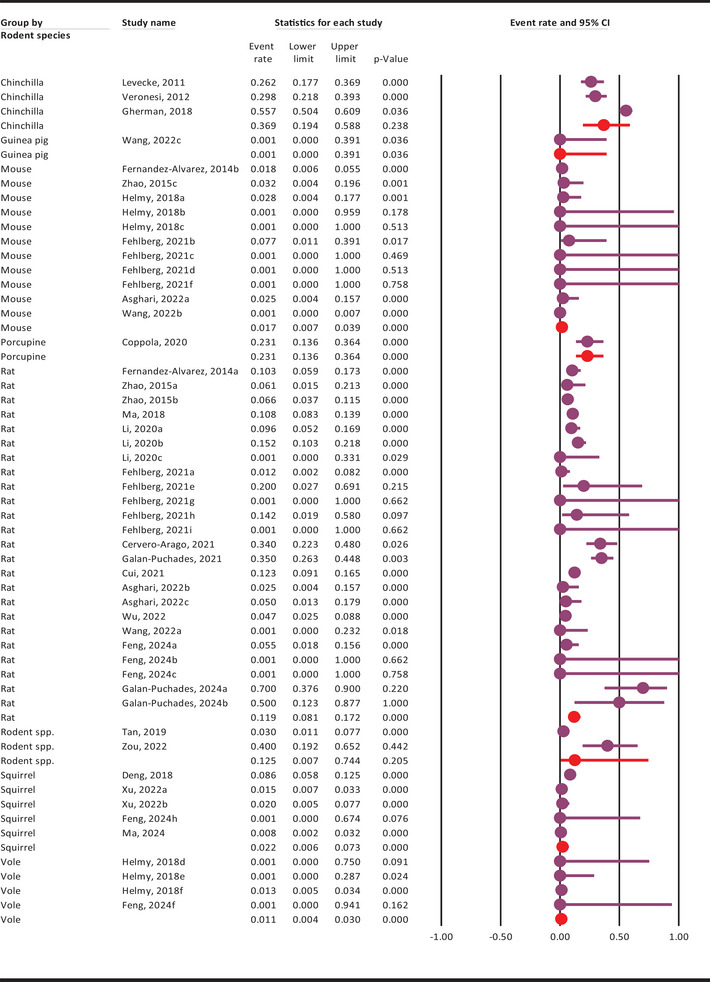
Pooled molecular prevalence of *G. duodenalis* in rodents by species, with 95% confidence intervals, estimated using a random‐effects model.

The subgroup analysis outcomes are presented in Table 1 and Supplementary Figures 1–4. In brief, the highest pooled molecular prevalence of *G. duodenalis* in rodents was observed between 2011 and 2014 at 13.1% (95% CI: 5.3–29.1%), while the lowest was reported from 2015 to 2019 at 5.3% (95% CI: 1.8–14.6%). Geographically, the highest pooled molecular prevalence occurred in rodents from Europe and the EUR WHO region, reaching 17.9% (95% CI: 9.8–30.5%) (Figure 4). Among evaluated countries, Romania (55.7%), Austria (34%), Italy (27.7%), Belgium (26.2%), and Spain (24%) reported the highest infection rates. Studies with fewer than 100 samples showed a higher prevalence (11.2%, 95% CI: 6.7–18.1%) compared to those with larger sample sizes (5.3%, 95% CI: 2.7–10.3%). Species‐specific data revealed the highest infection rates in chinchillas (36.9%) and porcupines (23.1%), though these findings are based on very limited datasets (Figure 5).

**TABLE 1:** Subgroup analysis of *G. duodenalis* molecular prevalence in rodents by publication year, continent, WHO region, country, sample size and rodent species.

Subgroup variable	Prevalence % (95% CI)	Heterogeneity (Q)	df (Q)	I^2^ (%)	*p*‐value
Publication year					
2011‐2014	13.1 (5.3‐29.1)	34.4	3	91.3	*p* < 0.05
2015‐2019	5.3 (1.8‐14.6)	340.7	12	96.5	*p* < 0.05
2020–2024	7.6 (4.5‐12.6)	207.3	33	84.1	*p* < 0.05
Continent					
Asia	5.1 (3.4‐7.5)	111	25	77.5	*p* < 0.05
Europe	17.9 (9.8‐30.5)	200.3	15	92.5	*p* < 0.05
South America	6.7 (2.5‐16.9)	5.4	8	0	*p* > 0.05
WHO region					
AMR	6.7 (2.5‐16.9)	5.4	8	0	*p* > 0.05
EMR	3.5 (1.3‐9)	0.5	2	0	*p* > 0.05
EUR	17.9 (9.8‐30.5)	200.3	15	92.5	*p* < 0.05
WPR	5.3 (3.5‐8)	106.9	22	79.4	*p* < 0.05
Country					
Austria	34 (22.3‐48)	0	0	0	*p* > 0.05
Belgium	26.2 (17.7‐36.9)	0	0	0	*p* > 0.05
Brazil	6.7 (2.5‐16.9)	5.4	8	0	*p* > 0.05
China ^a^	5.5 (3.6‐8.4)	101.8	21	79.4	*p* < 0.05
Germany	1.4 (0.6‐3.2)	2	5	0	*p* > 0.05
Iran	3.5 (1.3‐9)	0.5	2	0	*p* > 0.05
Italy	27.7 (21.2‐35.3)	0.8	1	0	*p* > 0.05
Malaysia	3 (1.1‐7.7)	0	0	0	*p* > 0.05
Romania	55.7 (50.4‐60.9)	0	0	0	*p* > 0.05
Spain	24 (9.1‐49.9)	42.6	4	90.6	*p* < 0.05
Sample size					
≤ 100	11.2 (6.7‐18.1)	103.7	32	69.1	*p* < 0.05
> 100	5.3 (2.7‐10.3)	509.8	17	96.7	*p* < 0.05
Rodent species					
Chinchilla	36.9 (19.4‐58.8)	35	2	94.3	*p* < 0.05
Guinea pig	0.1 (0‐39.1)	0	0	0	*p* > 0.05
Mouse	1.7 (0.7‐3.9)	12	10	16.6	*p* > 0.05
Porcupine	23.1 (13.6‐36.4)	0	0	0	*p* > 0.05
Rat	11.9 (8.1‐17.2)	115.6	23	80.1	*p* < 0.05
Rodent spp.	12.5 (0.7‐74.4)	17.6	1	94.3	*p* < 0.05
Squirrel	2.2 (0.6‐7.3)	26	4	84.6	*p* < 0.05
Vole	1.1 (0.4‐3)	1.3	3	0	*p* > 0.05

^a^ World Health Organization (WHO).

^b^ Region of the Americas (AMR).

^c^ Eastern Mediterranean Region (EMR).

^d^ European Region (EUR).

^e^ Western Pacific Region (WPR).

^f^ Due to CMA software limitations, a prevalence rate of 0.01% was used instead of 0% in calculations.

^g^ Of the 25 datasets from China, three were excluded from the meta‐analysis due to single‐sample size.

^h^ Rodents with unspecified species or ambiguous information were included in the group of rodent spp.

^i^ Chipmunk and marmot were included in the group of squirrels.

### 3.6 Assemblage/Sub‐Assemblage Distribution of *G. duodenalis* in Rodents

Among the eight reported *G. duodenalis* assemblages (A‐H), six assemblages (A‐E and G) and four sub‐assemblages (AI, AII, BIII, and BIV) were found in rodents. Zoonotic assemblage B and rodent‐specific assemblage G were the most frequently detected in rodents (Table 2 and Table 3).

**TABLE 2:** The main characteristics of 23 molecular studies (54 datasets) on the molecular prevalence and assemblage/sub‐assemblage distribution of *G. duodenalis* in rodents.

Author, year	Host's common name	Host's scientific name	Time tested	Country	Total no.	Infected no.	Prevalence (%)	Assemblages	Sub‐assemblages
Levecke, 2011	Long‐tailed chinchilla	*Chinchilla lanigera*	2007	Belgium	80	21	26.2	A, B, C, E	AI, AII, BIV, BIV/BIII
Veronesi, 2012	Long‐tailed chinchilla	*Chincilla lanigera*	2010	Italy	104	31	29.8	B, C	−
Fernandez‐Alvarez, 2014a	Black rat	*Rattus rattus*	2007–2011	Spain	116	12	10.3	G, B	BIV
Fernandez‐Alvarez, 2014b	House mouse	*Mus musculus*	2007–2011	Spain	165	3	1.8	G	−
Zhao, 2015a	Tanezumi rat	*Rattus tanezumi*	UC	China	33	2	6.1	G	−
Zhao, 2015b	Brown rat	*Rattus norvegicus*	UC	China	168	11	6.6	G	−
Zhao, 2015c	House mouse	*Mus musculus*	UC	China	31	1	3.2	G	−
Gherman, 2018	long‐tailed chinchilla	*Chinchilla lanigera*	UC	Romania	341	190	55.7	B, D, E	BIII, BIV
Deng, 2018	Chipmunk	*Eutamias asiaticus*	2016–2017	China	279	24	8.6	A, G	−
Helmy, 2018a	Striped field mouse	*Apodemus agrarius*	2011–2012	Germany	35	1	2.8	A	−
Helmy, 2018b	Yellow‐necked mouse	*Apodemus flavicollis*	2011–2012	Germany	38	0	0	−	−
Helmy, 2018c	Wood mouse	*Apodemus sylvaticus*	2011–2012	Germany	9	0	0	−	−
Helmy, 2018d	Short‐tailed field vole	*Microtus agrestis*	2011–2012	Germany	60	0	0	−	−
Helmy, 2018e	Common vole	*Microtus arvalis*	2011–2012	Germany	107	0	0	−	−
Helmy, 2018f	Bank vole	*Myodes glareolusv*	2011–2012	Germany	301	4	1.3	A, B	−
Ma, 2018	Bamboo rat	*Rhizomys sinensis*	2017	China	480	52	10.8	B	−
Tan, 2019	Rodent spp.	−	2011	Malaysia	134	4	3	B	−
Li, 2020a	Wistar rat	−	2019	China	104	10	9.6	G	−
Li, 2020b	Sprague‐dawley rat	−	2019	China	151	23	15.2	G	−
Li, 2020c	Spontaneously hypertensive rat	−	2019	China	100	0	0	−	−
Coppola, 2020	Crested porcupine	*Hystrix cristata*	2018–2019	Italy	52	12	23.1	A, B	AII, BIV
Fehlberg, 2021a	Rice rat	*Hylaeamys laticeps*	2015–2016	Brazil	81	1	1.2	A	AI
Fehlberg, 2021b	Cursor grass mouse	*Akodon cursor*	2015–2016	Brazil	13	1	7.7	A	AI
Fehlberg, 2021c	Atlantic forest climbing mouse	*Rhipidomys mastacalis*	2015–2016	Brazil	11	0	0	−	−
Fehlberg, 2021d	Blackish grass mouse	*Thaptomys nigrita*	2015–2016	Brazil	9	0	0	−	−
Fehlberg, 2021e	Rice rat	*Oecomys catherinae*	2015–2016	Brazil	5	1	20	A	AI
Fehlberg, 2021f	Caatinga vesper mouse	*Calomys expulsus*	2015–2016	Brazil	2	0	0	−	−
Fehlberg, 2021g	Rice rat	*Cerradomys subflavus*	2015–2016	Brazil	4	0	0	−	−
Fehlberg, 2021h	Rice rat	*Oligoryzomys nigripes*	2015–2016	Brazil	7	1	14.2	A	AI
Fehlberg, 2021i	Rice rat	*Euryoryzomys russatus*	2015–2016	Brazil	4	0	0	−	−
Cervero‐Arago, 2021	Brown rat	*Rattus norvegicus*	2017	Austria	50	17	34	A, G	AI, AII
Galan‐Puchades, 2021	Brown rat	*Rattus norvegicus*	2016–2017	Spain	100	35	35	−	−
Cui, 2021	Coypus/swamp rats	*Myocastor coypus*	UC	China	308	38	12.3	A, B	AI, BIV
Asghari, 2022a	House mouse	*Mus musculus*	2021	Iran	40	1	2.5	G	−
Asghari, 2022b	Brown rat	*Rattus norvegicus*	2021	Iran	40	1	2.5	G	−
Asghari, 2022c	Black rat	*Rattus rattus*	2021	Iran	40	2	5	B, G	−
Xu, 2022a	Himalayan marmot	*Marmota himalayana*	2017	China	399	6	1.5	A, B, E	−
Xu, 2022b	Alashan ground squirrel	*Spermophilus alashanicus*	2017	China	99	2	2	B	−
Wu, 2022	Brown rat	*Rattus norvegicus*	2010–2021	China	191	9	4.7	G	−
Wang, 2022a	Laboratory rat	−	2019–2020	China	118	0	0	−	−
Wang, 2022b	Laboratory mouse	−	2019–2020	China	1027	0	0	−	−
Wang, 2022c	Laboratory guinea pigs	−	2019‐2020	China	92	0	0	−	−
Zou, 2022	Rodent spp.	−	UC	China	15	6	40	−	−
Feng, 2024a	Brown rat	*Rattus norvegicus*	2018–2021	China	55	3	5.5	G, A	−
Feng, 2024b	Lesser ricefield rat	*Rattus losea*	2018–2021	China	4	0	0	−	−
Feng, 2024c	Greater bandicoot rat	*Bandicota indica*	2018–2021	China	2	0	0	−	−
Feng, 2024d	Chestnut white‐bellied rat	*Niviventer fulvescens*	2018–2021	China	1	0	0	−	−
Feng, 2024e	Plateau zokor	*Myospalax fontanieri*	2018–2021	China	1	0	0	−	−
Feng, 2024f	Yunnan red‐backed vole	*Eothenomys miletus*	2018–2021	China	41	0	0	−	−
Feng, 2024g	Pallas's squirrel	*Callosciurus erythraeus*	2018–2021	China	1	0	0	−	−
Feng, 2024h	Long‐tailed ground squirrel	*Spermophilus undulatus*	2018–2021	China	66	0	0	−	−
Ma, 2024	Himalayan marmot	*Marmota himalayana*	2017–2019	China	243	2	0.8	A	−
Galan‐Puchades, 2024a	Brown rat	*Rattus norvegicus*	2021–2023	Spain	10	7	70	−	−
Galan‐Puchades, 2024b	Black rat	*Rattus rattus*	2021–2023	Spain	4	2	50	−	−

^a^ This table includes only data related to *G. duodenalis* as confirmed in each study. For studies that examined multiple rodent species or datasets, only those in which *G. duodenalis* was specifically identified (typically through molecular methods) were included. If *Giardia* spp. was reported without species‐level confirmation in certain rodent species or datasets within the same study, those data were excluded from this table/study.

^b^ Some studies have performed molecular analysis on a portion of microscopically positive cases, rather than all samples. Hence, molecular prevalence was calculated based on the number of positive samples using only the molecular method.

^C^ Due to CMA software limitations, a prevalence rate of 0.01% was used instead of 0% in calculations, and three datasets from China with a total sample size of one were excluded from the analysis.

**TABLE 3:** Assemblage distribution of *G. duodenalis* in rodents by countries and rodent species.

Variables	Dataset no.	Total samples (no.)	Infected samples (no.)	Reported assemblages (no.)						
				A	B	C	D	E	G	ND
**Countries**										
Austria	1	50	17	9	−	−	−	−	3	5
Belgium	1	80	21	11	18	15	−	2	−	−
Brazil	9	136	4	4	−	−	−	−	−	−
China	25	4,009	189	19	94	−	−	1	69	6
Germany	6	550	5	3	2	−	−	−	−	−
Iran	3	120	4	−	1	−	−	−	3	−
Italy	2	156	43	2	41	2	−	−	−	−
Malaysia	1	134	4	−	1	−	−	−	−	3
Romania	1	341	190	−	151	−	33	6	−	−
Spain	5	395	59	−	1	−	−	−	14	44
**Total no**.	**54**	**5,971**	**536**	**48**	**309**	**17**	**33**	**9**	**89**	**58**
**Species**										
Chinchilla	3	525	242	11	198	17	33	8	−	−
Guinea pig	1	92	0	−	−	−	−	−	−	−
Mouse	11	1,380	7	2	−	−	−	−	5	−
Porcupine	1	52	12	2	12	−	−	−	−	−
Rat	26	2,177	227	15	90	−	−	−	73	49
Rodent spp.	2	149	10	−	1	−	−	−	−	9
Squirrel	6	1,087	34	16	6	−	−	1	11	−
Vole	4	509	4	2	2	−	−	−	−	−
**Total no**.	**54**	**5,971**	**536**	**48**	**309**	**17**	**33**	**9**	**89**	**58**

^a^ The plateau zokor was placed in the group of rats.

^b^ Rodents with unspecified species or ambiguous information were included in the group of rodent spp.

^c^ Chipmunk and marmot were included in the group of squirrels.

^d^ ND, Not determined.

^e^ Of the 25 datasets from China, three were excluded from the meta‐analysis due to single‐sample size.

^f^ When the number of assemblages exceeds the number of infected cases, mixed infections are present. Conversely, when the number of assemblages is less than the number of infected cases, this reflects either limited access to the full text or challenges in genotyping/sequencing.

### 3.7 Assemblages Distribution of *G. duodenalis* by Country and Rodent Species

In brief, China contributed the largest number of molecular datasets (*n* = 25) and rodent samples (*n* = 4,009). The greatest genetic diversity was observed in China (A, B, E, G) and Belgium (A, B, C, E), with assemblage B being the most prevalent. Assemblage D was found exclusively in Romania, while assemblage C was reported from Belgium and Italy. The highest assemblage diversity was observed in chinchillas (five: A–E), squirrels (four: A, B, E, G), and rats (three: A, B, G) (Table 3).

### 3.8 Assemblages Distribution of *G. duodenalis* by Continent

**FIGURE 6:**
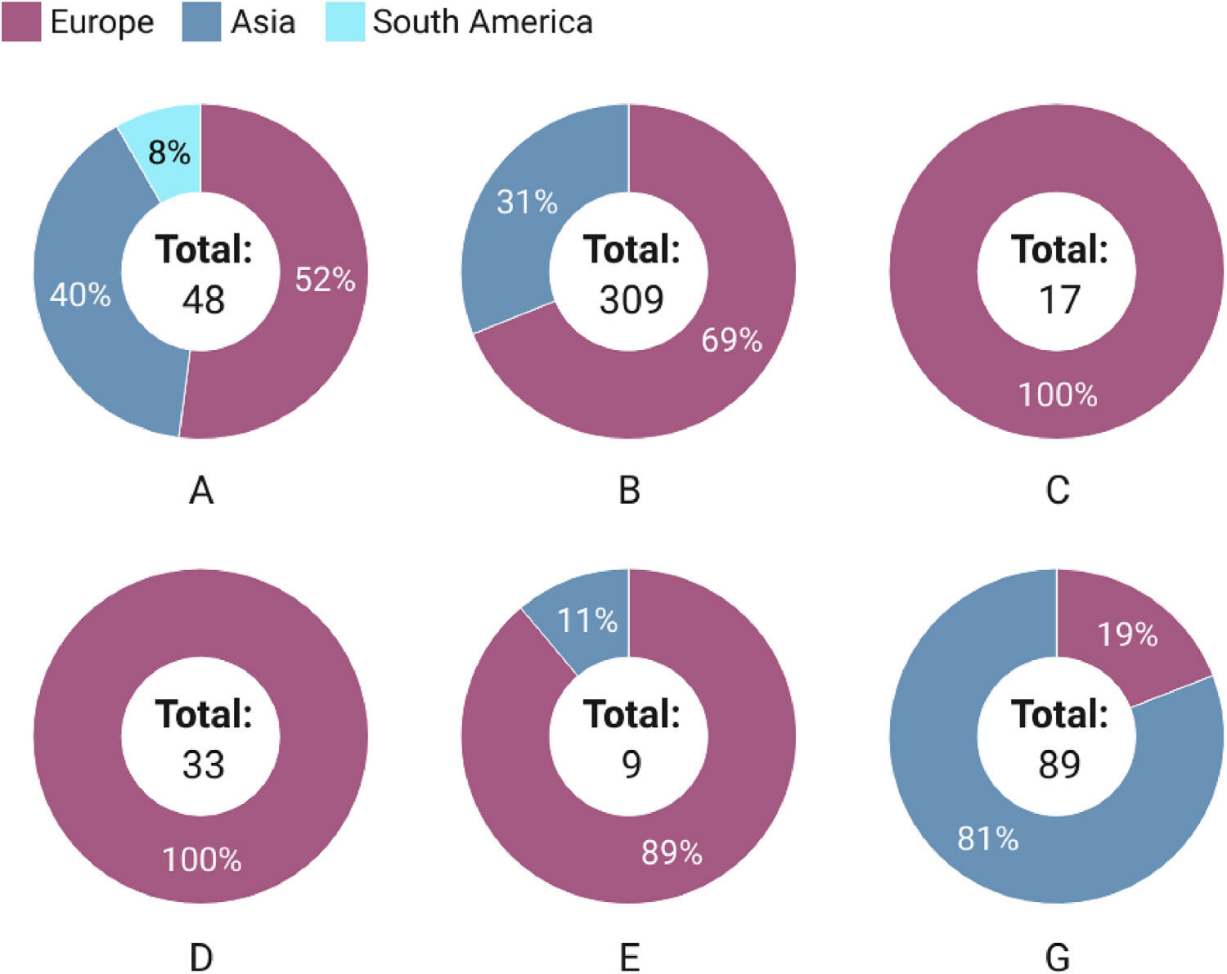
Assemblage distribution of *G. duodenalis* in rodents by continents. The numbers in the circle centres represent the total count of each assemblage isolated from rodents, while the percentages around the circles indicate the prevalence rate of each assemblage per continent.

In brief, assemblages C, D, G, and most occurrences of E, B, and A were reported from Europe; C and D were absent in Asia. In contrast, most reports of assemblage G originated from Asia, while only assemblage A was identified in South America (Brazil) (Figure 6).

## 4 Discussion

Prevalence‐based evaluations and the distribution of *G. duodenalis* assemblages/sub‐assemblages in rodents are essential for understanding the epidemiology of *G. duodenalis*, a protozoan parasite that affects both animal and human health (Helmy et al. 2018; Li et al. 2023). Hence, this systematic review and meta‐analysis aimed to determine the global molecular prevalence and genotypic distribution of *G. duodenalis* in rodents, based exclusively on studies using molecular diagnostic techniques.

Global meta‐analyses on the occurrence of *G. duodenalis* in animals are scarce. However, the prevalence of this parasitic protozoan has been reported as 22% (95% CI: 17–28%) in cattle (Taghipour et al. 2022), 15.2% (95% CI: 13.8–16.7%) in dogs, 12% (95% CI: 9.2–15.3%) in cats (Bouzid et al. 2015), and 9.1% (95% CI: 5.6–14.3%) in pigs (Asghari et al. 2023). Despite variations in study methods, geographical locations, and sample sizes, the reported prevalence of 7.4% of *G. duodenalis* in rodents is relatively low compared to other animals. The sensitivity analysis revealed no outlier data among the included studies that could significantly impact the final molecular prevalence of *G. duodenalis* in rodents. This finding underscores the robustness of the analysis and reinforces the reliability of the estimated prevalence rates.

Analysis of subgroup prevalence revealed no distinct trend in *G. duodenalis* infection rates among rodents over the years. The highest molecular prevalence was recorded between 2011 and 2014 at 13.1% (95% CI: 5.3–29.1%), while the lowest occurred from 2015 to 2019 at 5.3% (95% CI: 1.8–14.6%). Temporal shifts in infection rates may correlate with environmental factors and changes in rodent habitats. Rodents in Europe and the EUR WHO region exhibited the highest pooled molecular prevalence at 17.9% (95% CI: 9.8–30.5%). Notably, countries such as Romania (55.7%, 95% CI: 50.4–60.9%), Austria (34%, 95% CI: 22.3–48%), Italy (27.7%, 95% CI: 21.2–35.3%), Belgium (26.2%, 95% CI: 17.7–36.9%), and Spain (24%, 95% CI: 9.1–49.9%) reported the highest infection rates of *G. duodenalis* in rodents. European countries showed a higher pooled molecular prevalence of *G. duodenalis* in rodents, suggesting that varying ecological conditions and public health practices may affect its spread among rodent populations. Of note, most of these findings are derived from single studies or a limited number of investigations; therefore, they should be interpreted with caution. Based on sample size, the pooled molecular prevalence of *G. duodenalis* in rodents was 5.3% (95% CI: 2.7–10.3%) in studies with more than 100 samples, compared to 11.2% (95% CI: 6.7–18.1%) in studies with fewer than 100 samples. This difference in prevalence rates suggests that sample size has a significant impact on the observed prevalence. Larger sample sizes tend to provide more stable and reliable estimates, reducing the influence of random variation. The narrower confidence interval in studies with sample sizes over 100 further supports this notion. The higher molecular prevalence observed in smaller studies might be due to overestimation resulting from chance or specific characteristics of the limited number of rodents examined.

*G. duodenalis* showed the highest molecular prevalence in chinchillas (36.9%) and porcupines (23.1%). However, these findings are based on very limited data (three studies for chinchillas and one for porcupines), preventing any broader statistical or epidemiological conclusions. It remains unclear whether this reflects a true epidemiological trend or simply a sampling artefact. Some studies included only one or a few rodents per species, whereas others had sample sizes exceeding 1000 specimens, notably in China, which also reported the greatest assemblage diversity. This likely reflects a sampling bias, rather than true geographic variation in genotypic distribution.

Notably, of the eight reported *G. duodenalis* assemblages (A‐H) (Wang et al. 2014; Ryan et al. 2021), six assemblages (A‐E and G) and four sub‐assemblages (AI, AII, BIII, and BIV) have been found in rodents. Assemblage B and rodent‐specific assemblage G were the most frequently detected in rodents. Notably, the highest assemblage diversity was observed in chinchillas (five: A–E), squirrels (four: A, B, E, G), and rats (three: A, B, G). Sub‐assemblage AI was reported in both humans and animals, while sub‐assemblage AII is primarily associated with humans. Sub‐assemblages BIII and BIV were reported in humans as well as companion and wild animals (Mbae et al. 2016; Pipiková et al. 2020). Two of these assemblages, A and B, have significant zoonotic transmission potential and can lead to human infections and symptoms (Zajaczkowski et al. 2021). Although, there have been sporadic reports of human infection with other assemblages (Soliman et al. 2011; Zahedi et al. 2017; Pipiková et al. 2020). The zoonotic nature of assemblages A and B highlights the importance of understanding transmission dynamics in both rodent populations and human interactions with these animals. Surveillance efforts are crucial in regions where these assemblages are prevalent, as they can inform public health strategies aimed at reducing the risk of human infections. In addition, environmental factors such as habitat destruction and climate change may influence the distribution of *G. duodenalis* assemblages in wildlife, thereby affecting their potential to spill over into human populations. Of note, some analyses in this study rely on a limited number of studies/datasets, necessitating cautious interpretation.

This systematic review and meta‐analysis revealed considerable geographic variation in the prevalence and genotypic diversity of *G. duodenalis* assemblages among rodent populations across different countries. China, with the largest number of datasets (25) and the highest sample size (4,009 rodents), reported the greatest assemblage diversity (A, B, E, and G), while Belgium, despite having only a single study with a limited number of samples, also exhibited notable genetic diversity (A, B, C, and E). These findings suggest that the observed diversity in China may be influenced more by sampling intensity than by actual ecological variation, highlighting a potential sampling bias. In contrast, the unexpected diversity reported from countries with minimal data, such as Belgium, raises questions about the local transmission dynamics and host‐specific factors contributing to assemblage distribution. Assemblage B was the most frequently detected across most countries, although its prevalence varied widely, possibly reflecting differences in ecological conditions, rodent species, or methodological approaches. The occurrence of unique assemblages in specific regions (e.g., D in Romania, C in Belgium and Italy) further emphasises the need for more balanced and comprehensive sampling efforts to better understand the global distribution and epidemiological significance of *G. duodenalis* in rodent hosts.

Analysis of *G. duodenalis* assemblages across continents showed that assemblages C, D, G, and most occurrences of E, B, and A were reported from Europe; C and D were absent in Asia. In contrast, most reports of assemblage G originated from Asia, while only assemblage A was identified in South America (Brazil) (Fehlberg et al. 2021). Overall, the lack of data from North America, Oceania, and Africa indicates a significant gap in our understanding of *G. duodenalis* genetic diversity worldwide. Assemblages C and D, typically associated with canids (Adell‐Aledón et al. 2018), were identified in rodents from Europe but not from Asia. This geographic discrepancy may stem from regional differences in host species, environmental exposure, or diagnostic methodologies, but more plausibly reflects gaps in sampling rather than biological absence. Interestingly, most assemblage types (excluding the feline‐specific F and the pinniped‐associated H) were identified in rodents, yet the majority of studies did not report sub‐assemblage level data, which is essential for assessing zoonotic potential more precisely. Overall, much remains to be explored regarding the epidemiology and genetic diversity of *G. duodenalis* in rodents. As the number of studies increases across various geographic regions and rodent species, shifts in infection prevalence, changes in the distribution of assemblages and sub‐assemblages, and even the identification of novel assemblages, such as assemblage F, may be observed.

The health status of the sampled rodents was rarely reported, and there was little to no information on whether rodents were sampled randomly or due to clinical suspicion. This undermines the accuracy of reported prevalence rates and limits the ecological interpretation of the findings. Similarly, the habitat characteristics, immunological status, and interaction frequency with human populations were generally missing from the studies reviewed. These are critical factors in evaluating the real‐world risk of zoonotic transmission.

Preventive measures should focus on standardised monitoring of rodent populations in urban and peri‐urban areas, particularly where human‐animal interactions are frequent. Environmental surveillance, sanitation improvement, and rodent population control could reduce the potential risk of cyst contamination in water and food sources.

In light of the evidence, the role of rodents in the transmission cycle of human giardiasis should be considered limited but not negligible. Their capacity to act as reservoirs may be context‐dependent, influenced by local ecological and social determinants. However, current data are insufficient to support any broad claims regarding rodents as a significant source of human infection.

## 5 Conclusion

While the presence of zoonotic *G. duodenalis* assemblages in rodents is of scientific interest, the low overall prevalence, lack of consistent sampling protocols, unknown health status of hosts, and insufficient ecological context preclude a definitive conclusion about their significance as reservoirs for human infection. This review should be considered a preliminary exploration, a ‘tip of the iceberg’ of the potential role of rodents in giardiasis epidemiology. Future research should incorporate well‐designed epidemiological studies with large and ecologically diverse rodent populations, include randomised and stratified sampling, and utilise sub‐assemblage level genotyping. Stakeholders, including public health authorities, environmental agencies, and zoonotic disease surveillance units, should prioritise integrated One Health approaches, promoting cross‐sectoral collaboration to assess and mitigate the risks posed by wildlife reservoirs such as rodents.

## Author Contributions

Ali Asghari and Tahereh Davoodi planned and designed the study. Ali Asghari, Jalil Feizi, Kambiz Karimi, Mohammad Reza Mohammadi, Esfandiar Azizi, Zahra Bahramdoost, Ali Pouryousef and Fariba Shadfar were involved in the methodology and data extraction. Ali Asghari conducted the statistical analysis. Ali Asghari, Tahereh Davoodi, Jalil Feizi, and Fariba Shadfar wrote the manuscript and revised it. All authors have read and approved the final manuscript.

## Conflicts of Interest

The authors declare no conflicts of interest

## Peer Review

The peer review history for this article is available at https://www.webofscience.com/api/gateway/wos/peer‐review/10.1002/vms3.70546.

## Supporting information




**Supplementary Fig. 1**. The pooled molecular prevalence of G. duodenalis in rodents based on publication years.

**Supplementary Fig. 2**. The pooled molecular prevalence of G. duodenalis in rodents based on the WHO regions.

**Supplementary Fig. 3**. The pooled molecular prevalence of G. duodenalis in rodents based on countries.

**Supplementary Fig. 4**. The pooled molecular prevalence of G. duodenalis in rodents based on sample sizes.

**Supplementary Table 1**. JBI critical appraisal checklist applied for included studies

## Data Availability

The datasets used and/or analysed during the current study are available in the online version.
