# Supplementary material for: Giardia duodenalis in Rodents: A Global Systematic Review and Meta‐Analysis
Source: Vet Med Sci. 2025 Aug 12;11(5):e70546. doi: 10.1002/vms3.70546 (PMC12340709; doi:10.1002/vms3.70546)
Supplement: Supplementary file 4 — Supplementary Fig. 4. The pooled molecular prevalence of G. duodenalis in rodents based on sample sizes. [file VMS3-11-e70546-s001.docx]

**Supplementary Fig. 4.** The pooled molecular prevalence of *G. duodenalis* in rodents based on sample sizes.
