# Supplementary material for: Giardia duodenalis in Rodents: A Global Systematic Review and Meta‐Analysis
Source: Vet Med Sci. 2025 Aug 12;11(5):e70546. doi: 10.1002/vms3.70546 (PMC12340709; doi:10.1002/vms3.70546)
Supplement: Supplementary file 5 — Supplementary Table 1. JBI critical appraisal checklist applied for included studies [file VMS3-11-e70546-s003.docx]

**Supplementary Table 1**

**JBI critical appraisal checklist applied for included studies**

| Author Name/Year | Sample was representative? | Participants appropriately recruited? | Sample size was adequate? | Study subjects and the setting described? | Data analysis conducted | Objective, standard criteria, reliably used? | Appropriate statistical analysis used | Confounding factors/ subgroups/ differences identified and accounted? | Subpopulations identified using objective criteria | Overall quality |
| --- | --- | --- | --- | --- | --- | --- | --- | --- | --- | --- |
| Levecke, 2011 | Yes | Yes | Yes | Yes | Yes | Yes | Yes | Yes | No | 8/9 |
| Veronesi, 2012 | Yes | Yes | Yes | Yes | Yes | No | Yes | No | Yes | 7/9 |
| Fernandez-Alvarez, 2014 | Yes | No | Yes | Yes | Yes | Yes | Yes | No | Yes | 7/9 |
| Zhao, 2015 | Yes | No | Yes | Yes | No | Yes | No | No | No | 6/9 |
| Gherman, 2018 | Yes | Yes | Yes | Yes | Yes | No | Yes | Yes | No | 7/9 |
| Deng, 2018 | Yes | Yes | Yes | Yes | Yes | No | Yes | No | No | 6/9 |
| Helmy, 2018 | Yes | Yes | Yes | Yes | Yes | No | Yes | Yes | Yes | 8/9 |
| Ma, 2018 | Yes | Yes | Yes | Yes | Yes | Yes | Yes | No | No | 5/9 |
| Tan, 2019 | Yes | No | Yes | Yes | No | Yes | No | No | No | 6/9 |
| Li, 2020a | Yes | Yes | Yes | Yes | No | Yes | No | Yes | No | 6/9 |
| Coppola, 2020 | Yes | Yes | Yes | Yes | Yes | Yes | No | No | No | 4/9 |
| Fehlberg, 2021 | Yes | Yes | No | Yes | No | Yes | No | Yes | No | 5/9 |
| Cervero-Arago, 2021 | Yes | Yes | Yes | Yes | Yes | No | Yes | Yes | No | 7/9 |
| Galan-Puchades, 2021 | Yes | Yes | Yes | Yes | Yes | No | Yes | No | No | 6/9 |
| Cui, 2021 | Yes | Yes | Yes | Yes | Yes | No | Yes | Yes | Yes | 8/9 |
| Asghari, 2022 | Yes | Yes | Yes | Yes | Yes | Yes | Yes | Yes | No | 8/9 |
| Xu, 2022 | Yes | Yes | Yes | Yes | Yes | Yes | No | No | No | 4/9 |
| Wu, 2022 | Yes | No | Yes | Yes | No | Yes | No | No | No | 6/9 |
| Wang, 2022 | Yes | No | Yes | No | Yes | No | Yes | No | No | 4/9 |
| Zou, 2022 | Yes | Yes | No | Yes | No | Yes | No | Yes | No | 5/9 |
| Feng, 2024 | Yes | Yes | Yes | Yes | Yes | Yes | No | No | No | 6/9 |
| Ma, 2024 | Yes | Yes | Yes | Yes | Yes | Yes | No | No | No | 6/9 |
| Galan-Puchades, 2024 | Yes | Yes | Yes | Yes | Yes | Yes | Yes | No | No | 7/9 |
